# Newly Identified CYP2C93 Is a Functional Enzyme in Rhesus Monkey, but Not in Cynomolgus Monkey

**DOI:** 10.1371/journal.pone.0016923

**Published:** 2011-02-08

**Authors:** Yasuhiro Uno, Shotaro Uehara, Sakae Kohara, Kazuhide Iwasaki, Ryoichi Nagata, Koichiro Fukuzaki, Masahiro Utoh, Norie Murayama, Hiroshi Yamazaki

**Affiliations:** 1 Pharmacokinetics and Bioanalysis Center, Shin Nippon Biomedical Laboratories, Ltd., Kainan, Japan; 2 Business Development Department, Shin Nippon Biomedical Laboratories, Ltd., Osaka, Japan; 3 Laboratory of Drug Metabolism and Pharmacokinetics, Showa Pharmaceutical University, Machida, Japan; Dr. Margarete Fischer-Bosch Institute of Clinical Pharmacology, Germany

## Abstract

Cynomolgus monkey and rhesus monkey are used in drug metabolism studies due to their evolutionary closeness and physiological resemblance to human. In cynomolgus monkey, we previously identified cytochrome P450 (P450 or CYP) 2C76 that does not have a human ortholog and is partly responsible for species differences in drug metabolism between cynomolgus monkey and human. In this study, we report characterization of CYP2C93 cDNA newly identified in cynomolgus monkey and rhesus monkey. The CYP2C93 cDNA contained an open reading frame of 490 amino acids approximately 84–86% identical to human CYP2Cs. *CYP2C93* was located in the genomic region, which corresponded to the intergenic region in the human genome, indicating that *CYP2C93* does not correspond to any human genes. CYP2C93 mRNA was expressed predominantly in the liver among 10 tissues analyzed. The CYP2C93 proteins heterologously expressed in *Escherichia coli* metabolized human CYP2C substrates, diclofenac, flurbiprofen, paclitaxel, *S*-mephenytoin, and tolbutamide. In addition to a normal transcript (SV1), an aberrantly spliced transcript (SV2) lacking exon 2 was identified, which did not give rise to a functional protein due to frameshift and a premature termination codon. Mini gene assay revealed that the genetic variant IVS2-1G>T at the splice site of intron 1, at least partly, accounted for the exon-2 skipping; therefore, this genotype would influence CYP2C93-mediated drug metabolism. SV1 was expressed in 6 of 11 rhesus monkeys and 1 of 8 cynomolgus monkeys, but the SV1 in the cynomolgus monkey was nonfunctional due to a rare null genotype (c.102T>del). These results suggest that CYP2C93 can play roles as a drug-metabolizing enzyme in rhesus monkeys (not in cynomolgus monkeys), although its relative contribution to drug metabolism has yet to be validated.

## Introduction

The cytochrome P450 (*P450* or *CYP*) superfamily contains a large number of genes, 57 functional genes and 58 pseudogenes in human (see http://drnelson.uthsc.edu/cytochromeP450). One of its subfamilies, the CYP2C subfamily, comprising CYP2C8, CYP2C9, CYP2C18, and CYP2C19 in human, metabolizes approximately 20% of all prescribed drugs, including ibuprofen, phenytoin, tolbutamide, and warfarin [1]. Between human and mouse, the number of *CYP2Cs* differs, indicating the difficulty in determining orthologous relationships of *CYP2Cs* between the two species [2]. This suggests that the data from rodent must be cautiously interpreted and extrapolated to human.

For macaques, including cynomolgus monkey (*Macaca fascicularis*) and rhesus monkey (*Macaca mulatta*), the species used in preclinical studies, five CYP2C cDNAs have been identified in cynomolgus monkey and rhesus monkey, CYP2C8, CYP2C18, CYP2C43, CYP2C75, and CYP2C76 [3], [4], [5], [6], [7], [8]. After consulting with the P450 Nomenclature Committee (http://drnelson.uthsc.edu/cytochromeP450), in this paper, we designate cynomolgus monkey CYP2C20 and rhesus monkey CYP2C74, both orthologous to human CYP2C8, as CYP2C8. Macaque CYP2C8 shows 92% sequence identity to human CYP2C8, while macaque CYP2C43 and CYP2C75 are 91–93% identical to both human CYP2C9 and CYP2C19. In contrast, macaque CYP2C76 cDNA is only 70–72% identical to any human CYP2Cs [6], [8]. The investigation of CYP2C76 revealed that *CYP2C76* is not orthologous to human genes [6] and that CYP2C76 is at least partly responsible for the difference in drug metabolism between macaque and human [9]. Therefore, identification and characterization of the macaque-specific genes are important for understanding the differences of drug metabolism in the two species.

Macaques are essential for biomedical research of not only drug metabolism, but also neuroscience and behavior. Recently, the rhesus monkey genome has been sequenced [10] and now can be utilized to understand the similarities and differences between macaques and human in biomedical research. We have made extensive efforts to identify and characterize potential species-specific genes relevant to drug metabolism in macaque. In this paper, we report the identification of CYP2C93 in cynomolgus monkey and rhesus monkey. This novel CYP2C had a relatively low sequence identity to human and macaque CYP2Cs. For functional characterization of CYP2C93, mRNA expression was determined in various tissues and protein expression was analyzed in liver. Moreover, to assess orthologous relationships to human genes, the location of *CYP2C93* in the macaque *CYP2C* cluster was determined. Finally, the metabolic capacity of CYP2C93 was analyzed using heterologously expressed macaque CYP2C proteins and human CYP2C substrates (diclofenac, flurbiprofen, paclitaxel, *S*-mephenytoin, and tolbutamide). Further, the exon 2-skipped transcript variant identified in the course of this study was also characterized.

## Methods

### Ethics statement

All animal experiments (sample collection) were reviewed and approved by the Institutional Animal Care and Use Committee of Shin Nippon Biomedical Laboratories, Ltd. (approval no. PRF6043). All animals, housed and handled in strict accordance with good animal practice under supervision of veterinarians, received environmental enrichment and were monitored for evidence of disease and changes in attitude, appetite, or behavior suggestive of illness. In accordance with the recommendations of Weatherall report, “The use of non-human primates in research,” every effort was made to alleviate animal discomfort and pain by appropriate and routine use of anesthetic and/or analgesic agents.

### Materials

Diclofenac, flurbiprofen, paclitaxel, *S*-mephenytoin, and tolbutamide were purchased from Sigma-Aldrich (St. Louis, MO). Oligonucleotides and TaqMan probes were synthesized by Invitrogen (Tokyo, Japan) and Biosearch Technology Japan (Tokyo, Japan), respectively. All other reagents were purchased from Wako (Osaka, Japan) unless otherwise specified.

### Tissue samples and preparation of RNA and genomic DNA

Tissue samples were collected from 6 cynomolgus monkeys (3 males and 3 females from Indochina, 4–5 years of age, weighing 3–5 kg) and total RNA was extracted from the tissues as previously described [6]. The tissue samples collected were brain, lung, heart, liver, kidney, adrenal gland, jejunum, testis, ovary, and uterus, which were used to analyze the tissue expression pattern of CYP2C93 mRNA. Similarly, total RNA was also extracted from liver samples of 11 rhesus monkeys (5 males and 6 females from China, 7 years of age, weighing 3–5 kg) and 8 cynomolgus monkeys (from Indochina or Indonesia, 4–5 years of age, weighing 3–5 kg), which were used to analyze expression of CYP2C93 transcript variants. For the same animals, genomic DNA was extracted from whole blood samples using the PUREGENE DNA isolation kit (Gentra Systems, Minneapolis, MN) or from liver samples using the DNeasy Blood & Tissue kit (QIAGEN, Valencia, CA) according to the manufacturer's instructions.

### Cloning and sequencing

Reverse transcription (RT)-polymerase chain reaction (PCR) was performed as previously described [6] using total RNA extracted from cynomolgus monkey (mfF1) and rhesus monkey (mm35) liver. Briefly, the first-strand cDNA was generated in a mixture containing 1 µg of total RNA, oligo (dT), and SuperScript II RT reverse transcriptase (Invitrogen) at 37°C for 1 h. The subsequent PCR was carried out with the generated cDNA as a template using KOD Plus DNA polymerase (Toyobo, Osaka, Japan) according to the manufacturer's protocol with a thermal cycler (Applied Biosystems, Foster City, CA). PCR conditions include an initial denaturation at 95°C for 2 min and 35 cycles at 95°C for 20 s, 58°C for 20 s, and 72°C for 2 min, followed by a final extension at 72°C for 10 min. The primers used were 5′-ATGTCTGGAGAAGAGAAGGC-3′ and 5′-GACTTGCAGGTGACAAAAGATCA-3′. The amplified cDNAs were, after addition of an A-overhang, cloned into pCR2.1-TOPO vectors using TOPO TA Cloning Kit (Invitrogen) and the inserts were sequenced using ABI PRISM BigDye Terminator v3.0 Ready Reaction Cycle Sequencing Kit (Applied Biosystems), followed by electrophoresis with an ABI PRISM 3730 DNA Analyzer (Applied Biosystems).

### 5′ rapid amplification of cDNA ends

To verify the translational initiation codon of the cDNA sequence, 5′ rapid amplification of cDNA ends (RACE) was carried out with liver total RNA of cynomolgus monkey (mfF1) and rhesus monkey (mm35) using 5′ RACE System for Rapid Amplification of cDNA Ends (Invitrogen) according to the manufacturer's protocol. The primers for 5′ RACE were as follows: 5′-CAACTCCTCCACAATAC-3′ for RT reaction, 5′-CGCAGTCCTCAATGCTTCTCTTA-3′ for the initial PCR, and 5′-CCAAAATTTCGCAAGGTCAAA-3′ for the nested PCR. The PCR products were cloned into pCR2.1-TOPO vectors and the inserts were sequenced as described earlier.

### Analysis of CYP2C93 transcripts

Expression of CYP2C93 transcript variants (SV1 and SV2), retaining and lacking exon 2, respectively, was assessed by RT-PCR with additional liver total RNAs of 7 cynomolgus monkeys and 10 rhesus monkeys. RT products generated as described earlier were used for PCR that was performed with gene-specific primers (10 pmol), 0.2 mM dNTPs, 2 mM MgCl_2_, and 1 unit of AmpliTaq Gold DNA polymerase (Applied Biosystems) in a total volume of 20 µl. PCR conditions were as follows: 95°C for 10 min; 30 cycles at 95°C for 15 s, 60°C for 30 s, and 68°C for 1 min; final extension at 68°C for 5 min. The primers used were 5′-TCCTTTCACTCTGGAGACAGAGTTC-3′ and 5′-CCAAAATTTCGCAAGGTCAAA-3′. The reaction was run on an agarose gel to visualize the amplicons.

### Sequence analysis

Sequence data were analyzed with DNASIS Pro (Hitachi Software, Tokyo, Japan) and the Genetyx system (Software Development, Tokyo, Japan). Multiple alignment of amino acid sequences was performed using the ClustalW program and the results were used to create a phylogenetic tree by the neighbour-joining method. A homology search was conducted using BLAST (National Center for Biotechnology Information). The human, chimpanzee, orangutan, rhesus monkey, and marmoset genome data were analyzed using BLAT (UCSC Genome Bioinformatics). The P450 amino acid sequences found in GenBank were used for the analysis, including human CYP2A6 (NP_000753), CYP2C8 (NP_000761), CYP2C9 (NP_000762), CYP2C18 (NP_000763), and CYP2C19 (NP_000760); cynomolgus monkey CYP2C8 (P33262), CYP2C18 (ABB87194), CYP2C43 (AAZ29452), CYP2C75 (AAZ29451), and CYP2C76 (AAZ29453); rhesus monkey CYP2C8 (NP_001035300), CYP2C43 (NP_001035329), CYP2C75 (NP_001035301), and CYP2C76 (NP_001171259); marmoset CYP2C8 (BAF37097); dog CYP2C21 (AAC05209) and CYP2C41 (NP_001003334); and rat CYP2C6 (P05178), CYP2C7 (NP_058854), CYP2C11 (NP_062057), CYP2C12 (NP_113760), CYP2C13 (NP_612523), CYP2C22 (NP_612521), and CYP2C23 (NP_114027). Amino acid sequences deduced from the cynomolgus and rhesus monkey CYP2C93 cDNAs identified in this study were also used for the analysis. A one-base deletion in exon 1 of the cynomolgus monkey CYP2C93 cDNA was filled in with thymine, the nucleotide found in wild-type animals.

### Analysis of *CYP2C93* genomic arrangement

Analysis of bacterial artificial chromosome (BAC) clones was performed using the rhesus monkey *CYP2C* BAC clones (BACPAC Resources, Oakland, CA) as previously described [6] with the following modifications. PCR amplification was performed using the *CYP2C* BAC DNA as a template with gene-specific primers (5 pmol), 0.5 mM dNTPs, 2 mM MgCl_2_, and 1 unit of AmpliTaq Gold DNA polymerase (Applied Biosystems) in a total volume of 20 µl. PCR conditions were as follows: 95°C for 10 min; 30 cycles at 95°C for 20 s, 55°C for 20 s, and 72°C for 1 min; and a final extension at 72°C for 10 min. The *CYP2C93* primers used were; 5′-CTGGTGCTCTGTCTCTCCTGTT-3′ and 5′-TGCTGAAAGATTTGCTGATATTC-3′ for the 5′ end, and 5′-CTGAAATCTCTTACTGATTTAAAGGC-3′ and 5′-TCTGGTATGAAGGTAGCATAGAAACAAG-3′ for the 3′ end of the gene. The primers for *CYP2C8* and *CYP2C76* were the same as previously described [6].

### Amplification of *CYP2C93* exons

The gene fragments containing exon 1 or exons 2/3 were amplified for identification of genetic polymorphisms. Exon 6 was amplified to complete the *CYP2C93* sequence because the gene sequence around exon 6 was not found in the rhesus monkey genome data. PCR was carried out with cynomolgus monkey genomic DNA using 5 pmol of each primer, 0.5 mM dNTPs, 2 mM MgCl_2_, and 1 unit of LA Taq DNA polymerase (Takara, Tokyo, Japan) in a total volume of 20 µl. The primers used were 5′-CCTTGACTCCAATCCAATGC-3′ and 5′-CCAAAATGTTTCTCCACTCACA-3′ for exon 1, and 5′-GTTCTCCTGACCTCCGTTTC-3′ and 5′-CTGGAACCCAGGTTTATGCT-3′ for exons 2/3, and 5′-GACCTTCCCAGGCTTCAG-3′ and 5′-TCCTATTTTGGCAAACACCA-3′ for exon 6. Thermal cycler conditions were as follows: 95°C for 2 min; 35 cycles at 95°C for 20 s, 55°C for 30 s, and 72°C for 5 min; and a final extension at 72°C for 20 min. The PCR products were cloned into pCR-XL-TOPO vectors using TOPO XL Cloning Kit (Invitrogen) according to the manufacturer's protocol, followed by sequencing of the inserts as described earlier.

### Real-time RT-PCR

The expression level of the CYP2C93 mRNA was measured as previously described [6] using the primers and a probe specific for CYP2C93 mRNA with the following modifications. Briefly, the RT reaction was carried out using random primers (Invitrogen) as described above; one twenty-fifth of the volume was used for the subsequent PCR. The amplification was carried out in a total volume of 25 µl using TaqMan Universal PCR Master Mix (Applied Biosystems) with an ABI PRISM 7500 sequence detection system (Applied Biosystems) according to the manufacturer's protocol. The primers employed were 5′-GAGTGGCAACTTTAAGAAAAGTGAAA-3′ and 5′-TCTGGTATGAAGGTAGCATAGAAACAAG-3′, and the probe was 5′-FAM-CTCAATGCCACTCCCACTGCCAAA-BHQ-3′. The final concentration of the primer set and the probe was 600 and 200 nM, respectively. The primers and probes of CYP2C8, CYP2C43, CYP2C75, and CYP2C76 mRNAs were as described previously [6]. The relative expression level was determined by normalization of the raw data to the 18S ribosomal RNA level based on three independent amplifications.

### Heterologous protein expression in *Escherichia coli*

For characterization of CYP2C93 proteins, CYP2C93v1 and CYP2C93v2, corresponding to CYP2C93 transcript variants, SV1 and SV2, respectively, expression plasmids were generated and the proteins were expressed as previously reported [6], [11]. A total of three expression plasmids were constructed based on cynomolgus monkey CYP2C93 SV1 and SV2 cDNAs, and rhesus monkey CYP2C93 SV1 cDNA. CYP2C93 SV1 and SV2 cDNAs isolated from cynomolgus monkey contained a one-base deletion (c.102T>del); therefore c.102 was filled in with thymine, using the QuikChange XL II kit (Stratagene) according to the manufacturer's protocol, to generate plasmids for cynomolgus monkey CYP2C93. The primers used were 5′-CCTGGCCCCACtCCTCTCCCTATTATTGG-3′ and 5′-CCAATAATAGGGAGAGGaGTGGGGCCAGG-3′ where the lower case letters indicate the nucleotides to be altered. The insert sequences were confirmed by sequencing. Using these plasmids as templates, PCR was performed to modify the N-terminus of the expressed protein to enhance protein expression as described previously [6]. The PCR primers used were 5′-GGAATTCCATATGGCTCTGTTATTAGCAGTTTTTCTCTGTCTCTCCTGTTTGCTTCT-3′ and 5′-GCTCTAGACTTAACCTTCTTCAGACAGGAGT-3′. Expression plasmids of cynomolgus monkey CYP2C8, CYP2C43, CYP2C75, and CYP2C76 proteins were prepared as described previously [6]. Protein expression in *E. coli* using the expression plasmids and membrane preparations from the *E. coli* cell were performed as described previously [6], [11]. The content of P450 protein and NAPDH-P450 reductase in each membrane preparation was determined as described previously [11].

### Enzyme assays

Cynomolgus and rhesus monkey CYP2C93 proteins, along with cynomolgus monkey CYP2C8, CYP2C43, CYP2C75, and CYP2C76 proteins, were analyzed for drug-metabolizing capability using human CYP2C substrates, diclofenac, flurbiprofen, paclitaxel, *S*-mephenytoin, and tolbutamide, as described previously [12], [13], [14]. Briefly, a typical incubation mixture (0.25 ml) contained recombinant CYP2C protein (5 pmol) or cynomolgus monkey liver microsomes (0.1 mg protein/ml), an NADPH-generating system (0.25 mM NADP^+^, 2.5 mM glucose 6-phosphate, and 0.25 unit/ml glucose 6-phosphate dehydrogenase), and substrate (50 µM diclofenac, 100 µM flurbiprofen, 100 µM paclitaxel, 200 µM *S*-mephenytoin, or 1 mM tolbutamide) in 50 mM potassium phosphate buffer (pH 7.4). Reactions were incubated at 37°C for 15 min and terminated by adding 0.25 ml of ice-cold acetonitrile. After centrifugation at 900 *g* for 5 min, metabolites from diclofenac and flurbiprofen in the supernatant were determined by high-performance liquid chromatography with ultraviolet and fluorescence detectors, respectively. Metabolites of other substrates were determined, after extraction with ethyl acetate and evaporation to dryness, by high-performance liquid chromatography with an ultraviolet detector. For kinetic analysis, each substrate (0–5000 µM tolbutamide, 0–200 µM diclofenac, and 0–200 µM flurbiprofen) was incubated with recombinant CYP2C93v1 at 37°C for 15 min in the presence of an NADPH-generating system as described above. Kinetic parameters were calculated from a fitted curve by non-linear regression (mean ± SE).

### Minigene experiments

To characterize the genetic variant (IVS2-1G>T) possibly responsible for the exon 2-skipping, mini gene plasmids were constructed for *in vitro* splicing analysis. The *CYP2C93* gene fragment from exon 1 to exon 3 was amplified by PCR using the genomic DNAs derived from cynomolgus monkey (mfF1) expressing CYP2C93 SV1 and SV2, and rhesus monkey (mm35) expressing CYP2C93 SV1. PCR and subcloning into pCR-XL-TOPO vectors were performed as described above for amplification of exon 6. The PCR primers used were 5′-CCTTGACTCCAATCCAATGC-3′ and 5′-CTGGAACCCAGGTTTATGCT-3′. The resultant pCR-XL-TOPO plasmids contained either the *CYP2C93* gene fragment of the cynomolgus monkey with IVS2-1G or IVS2-1T, or the *CYP2C93* gene fragment of the rhesus monkey with IVS2-1G. To obtain the *CYP2C93* gene fragment of the rhesus monkey with IVS2-1T, the mutation IVS2-1G>T was introduced into the *CYP2C93* gene fragment of the rhesus monkey with IVS2-1T in the pCR-XL-TOPO plasmid, using the QuikChange XL II kit (Stratagene) according to the manufacturer's instructions. The primers used were 5′-CTCCTTTCCCAtTTCTCAAAACTC-3′ and 5′-GAGTTTTGAGAAaTGGGAAAGGAG-3′ where lower case letters indicate the nucleotides to be changed. Next, each pCR-XL-TOPO plasmid containing either of these four *CYP2C93* gene fragments was used as a template for PCR that was performed using KOD Plus DNA polymerase as described earlier, except that the annealing step was performed at 65°C for 20 s. The PCR primers used were 5′-CACCTAAGAAGAGAAGGCTTCAATGG-3′ and 5′-TCAATTGGTTTTTCTCAACTCCTCCA-3′. The PCR products were cloned into pcDNA3.1D/H5-His-TOPO vectors using the pcDNA3.1 Directional TOPO Expression Kit (Invitrogen) according to the manufacturer's instructions. Each plasmid insert was verified by sequencing. The resultant plasmids contained the *CYP2C93* gene fragment of the cynomolgus monkey with IVS2-1G (pcDNA3.1-mfCYP2C93_IVS2-1G) or IVS2-1T (pcDNA3.1-mfCYP2C93_IVS2-1T), or the *CYP2C93* gene fragment of the rhesus monkey with IVS2-1G (pcDNA3.1-mmCYP2C93_IVS2-1G) or IVS2-1T (pcDNA3.1-mmCYP2C93_IVS2-1T). For *in vitro* splicing analysis, these mini gene constructs were transfected into COS1 cells (2 × 10^5^ cell/well) (Riken, Tsukuba, Japan) using FuGene (Roche Applied Science, Indianapolis, IN) according to the manufacturer's protocol. At 24 h after transfection, cells were harvested and total RNA was extracted from the cells using the RNeasy Mini Kit (QIAGEN) according to the manufacturer's protocol. To identify CYP2C93 SV1 and SV2, RT-PCR was carried out with the extracted total RNA as described earlier for the analysis of CYP2C93 transcripts. β-actin, as a control, was also amplified by RT-PCR using the primers, 5′-AACGGTGAAGGTGACAGCA-3′ and 5′-AGTGGGGTGGCTTTTAGGA-3′. The PCR reaction was run on an agarose gel to visualize the amplified products.

### Genotyping

To genotype c.102T>del, the method was established with GeneMapper (Applied Biosystems). PCR was performed as described for analysis of CYP2C93 transcripts. The gene-specific primers synthesized (Applied Biosystems), 5′-CTGGTGCTCTGTCTCTCCTGTT-3′ and 5′-TGCTGAAAGATTTGCTGATATTC-3′, were used. The PCR products were electrophoretically analyzed with an ABI PRISM 3730 DNA Analyzer and were scored and genotyped using GeneMapper software (Applied Biosystems). The presence of c.102T>del was indicated by a peak size different from that of the wild-type genome. To genotype IVS2-1G>T, direct sequencing was carried out, by performing PCR as described above for amplification of *CYP2C93* exons.

## Results

### Identification of CYP2C93 cDNAs

**Figure 1:**
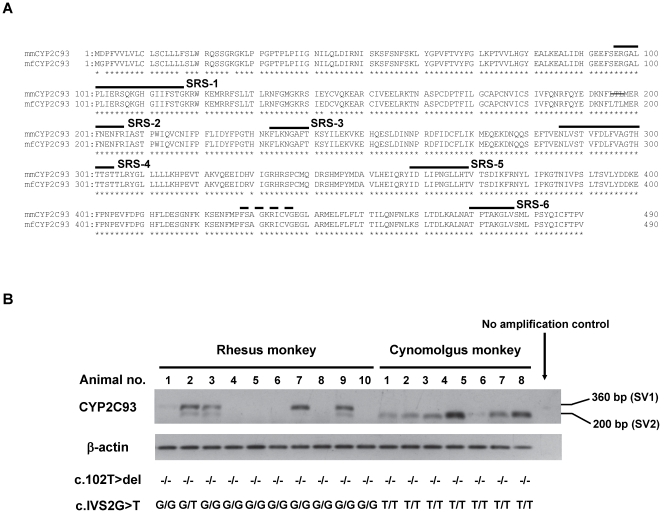
CYP2C93 amino acid sequences and hepatic expression of CYP2C93 transcripts. (**A**) Multiple alignment of cynomolgus monkey (mf) and rhesus monkey (mm) CYP2C amino acid sequences. For cynomolgus monkey CYP2C93, the amino acid sequences were predicted from transcript variant SV1 cDNA (c.102T was filled in with thymine). The broken and solid lines above the sequences indicate the putative heme-binding region and the six putative substrate recognition sites (SRSs), respectively. Asterisks under the sequences indicate identical amino acids. (**B**) Hepatic expression of two CYP2C93 transcript variants in cynomolgus monkeys and rhesus monkeys. Expression of normal transcript SV1 and aberrant transcript SV2 was analyzed in livers of 7 cynomolgus monkeys and 10 rhesus monkeys by RT-PCR and gel electrophoresis of the PCR products. SV1 was expressed in 5 rhesus monkeys, including animal number 1 which expressed SV1 faintly, whereas only SV2 was expressed in the cynomolgus monkeys. For these animals, genotyping of c.102T>del and IVS2-1G>T was determined. None of these animals possessed c.102T>del. All the cynomolgus monkeys were homozygous for IVS2-1G>T, but only one rhesus monkey possessed this allele (as a heterozygote).

A BLAST search of GenBank database using the human CYP2C9 cDNA sequence identified the CYP2C-like cDNA sequence, which was predicted by computer analysis based on rhesus monkey genome data. For cloning of this potential cDNA, RT-PCR was performed with liver total RNA of rhesus monkey (mm35) using the primers that were designed to amplify the putative open reading frame (ORF), which led to the successful identification of the cDNA for this novel CYP2C named CYP2C93 (Fig. 1A). Although the forward primer used for cloning the cDNA overlapped the putative translation initiation codon, the 5′RACE using liver total RNAs of cynomolgus monkey and rhesus monkey confirmed this translation initiation codon. The rhesus monkey CYP2C93 cDNA contained the ORF of 490 amino acids with primary sequence structures common to CYP2Cs including six potential substrate recognition sites (SRSs) [15] and heme-binding region (Fig. 1A). Amino acids deduced from this CYP2C93 cDNA had relatively low sequence identity (77-81%) to human, cynomolgus monkey, and rhesus monkey CYP2C proteins (Table 1).

**Table 1:** Sequence identity of CYP2C93 cDNA and amino acids as compared to human and other macaque CYP2Cs.

	cDNA		Amino acids
		%	
**Human:**			
CYP2C8	86		79
CYP2C9	85		77
CYP2C18	85		77
CYP2C19	84		79
**Cynomolgus:**			
CYP2C8	87		81
CYP2C43	83		77
CYP2C75	84		76
CYP2C76	77		71
**Rhesus:**			
CYP2C43	83		77
CYP2C74	87		81
CYP2C75	84		76
CYP2C76	77		71

In cynomolgus monkey, RT-PCR using liver total RNA of cynomolgus monkey (mfF1) gave rise to two CYP2C93 cDNAs of different sizes. Sequencing and analysis of these cDNAs showed that these two cDNAs corresponded to the CYP2C93 transcripts retaining (SV1) and lacking (SV2) exon 2, both nearly identical (>99%) to the rhesus monkey CYP2C93 cDNA sequence. Furthermore, CYP2C93 SV1 and SV2 contained a 1-base deletion (c.102T>del) in exon 1, which would generate frameshift and a premature termination codon. To identify the animals expressing SV1, RT-PCR was performed to amplify the cDNA fragments of exons 1–3 using additional liver samples from 7 cynomolgus monkeys (nos. 1–7), along with 10 rhesus monkeys (nos. 1–10). This analysis showed that all 7 cynomolgus monkeys expressed only SV2, whereas 5 of the 10 rhesus monkeys expressed SV1 (Fig. 1B). It should be noted that a faint band of SV1 was seen in rhesus monkey no. 1. In all the rhesus monkeys expressing SV1, SV2 was barely detectable on the gel. The sequences of the CYP2C93 cDNAs identified have been deposited in GenBank under the accession numbers, GU289739 (cynomolgus monkey SV1), GU289740 (cynomolgus monkey SV2), and GU289738 (rhesus monkey SV1).

**Figure 2:**
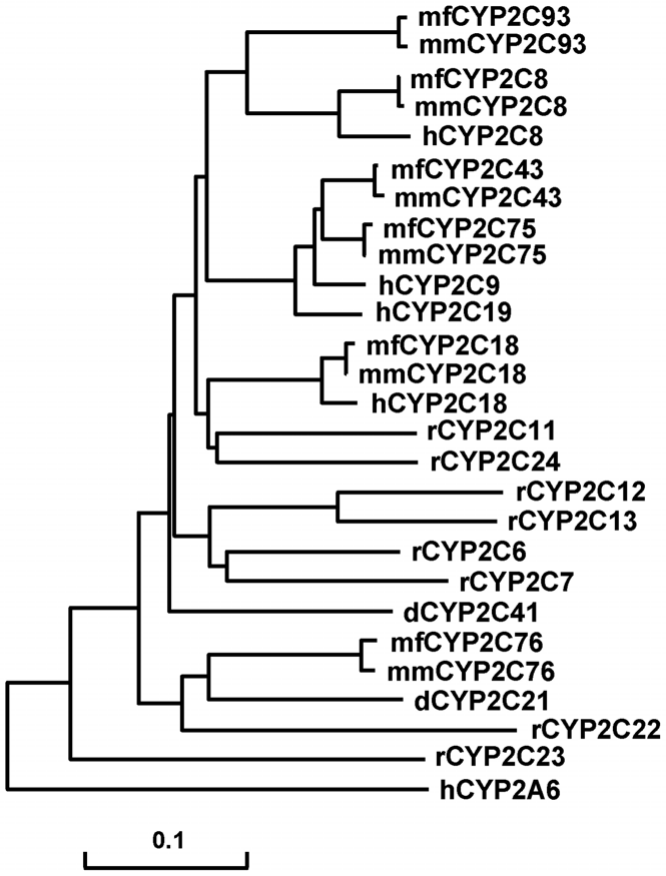
Phylogenetic tree of CYP2C amino acid sequences. The phylogenetic tree was created by the neighbour-joining method using CYP2C amino acid sequences of human (h), cynomolgus monkey (mf), rhesus monkey (mm), dog (d), and rat (r), found in GenBank. For cynomolgus monkey CYP2C93, the amino acid sequence was predicted from the SV1 cDNA (c.102 was filled in with thymine). Human CYP2A6 amino acid sequence was used as outgroup.

A phylogenetic tree of CYP2C amino acid sequences from human, cynomolgus monkey, rhesus monkey, dog, and rat indicated that CYP2C93 is orthologous between cynomolgus monkey and rhesus monkey, but does not have a 1-to-1 relationship to human CYP2C (Fig. 2).

### Genomic location of *CYP2C93* in the *CYP2C* cluster

**Figure 3:**
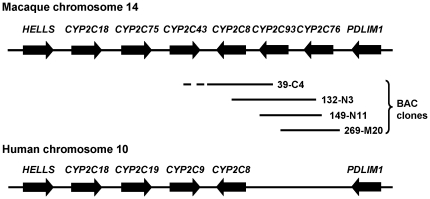
Genomic structure of the macaque *CYP2Cs*. The location and direction of *CYP2C8*, *CYP2C76*, and *CYP2C93* in the macaque *CYP2C* cluster were determined by PCR-amplification patterns using the macaque *CYP2C* BAC clones, and by the BLAT analysis of the rhesus monkey genome data. *CYP2C93*, along with *CYP2C76*, was located at the end of the gene cluster, the location of which corresponds to the intergenic region in the human genome.

To determine the genomic location of *CYP2C93*, PCR was performed using gene-specific primers with the rhesus monkey *CYP2C* BAC clones as templates. The amplification patterns indicated that *CYP2C93* was located adjacent to *CYP2C76* at the end of the *CYP2C* cluster. This location corresponds to the intergenic region in the human genome (Fig. 3). The BLAT analysis of the rhesus monkey genome mapped *CYP2C93* to the same location in the cluster, supporting the results obtained by our analysis of BAC clones. The BLAT analysis of the human, chimpanzee, orangutan, and marmoset genomes indicated that the genome sequences highly identical (≥90%) to CYP2C93 cDNA were not present in these genomes. These results suggest that a *CYP2C93* ortholog is not present in the human, chimpanzee, orangutan, and marmoset genomes.

### Gene structure of *CYP2C93*

The exon-intron structure was analyzed for *CYP2C93* by aligning rhesus monkey CYP2C93 cDNA on the rhesus monkey genome using BLAT. The sequence around exon 6 missing in the genome data was determined by PCR and sequencing. The analysis of the rhesus monkey genome data and the gene sequence containing exon 6 revealed that *CYP2C93* spanned approximately 25.6 kb and contained nine exons similar to human and other cynomolgus and rhesus monkey *CYP2Cs*. The sizes of exons and introns ranged from 141 to ≥244 bp and from 161 to 11401 bp, respectively (Table 2). All exons were flanked by GU and AG dinucleotides, consistent with the consensus sequences for splice junctions in eukaryotic genes.

**Table 2:** Exon-intron boundary sequences of *CYP2C93*.

Exon	Exon size	3′ splice site	5′ splice site	Intron size
	bp			bp
1	184		CAGCAAT**gt**aagtatg	1233
2	153	ctttccc**ag**TTCTCAA	GGGCATG**gt**aggtgtg	161
3	150	ttttgtt**ag**GAATCAT	ACCAATG**gt**gagtgac	2228
4	161	atccttt**ag**CCTCACC	GATCCAG**gt**gaggcca	1568
5	177	ttttttt**ag**GTTTGCA	GGAGCAG**gt**aagatgt	11401
6	141	tatttct**ag**GAAAAGG	GTCACAG**gt**aggacca	3226
7	189	tcttatc**ag**CTAAAGT	CCCTAAG**gt**aagcttg	3070
8	142	tacttcc**ag**GGCACAA	TCAGCAG**gt**aatggaa	2026
9	≥244	tattttc**ag**GAAAACGG		

Exon and intron sequences are indicated in capital and lower case letters, respectively.

The dinucleotide sequence at the highly conserved GU-AG motif is shown as underlined bold lettering.

### CYP2C93 mRNA expression in tissues

**Figure 4:**
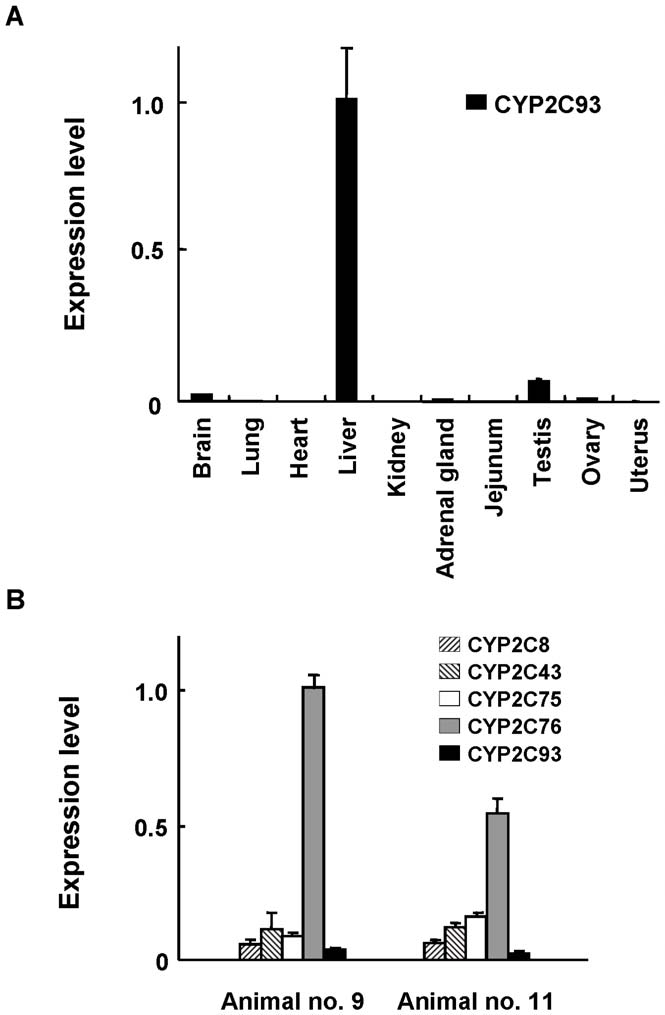
Measurement of CYP2C93 mRNA tissue expression. Real-time RT-PCR was performed using the probe and primer set specific for CYP2C8, CYP2C43, CYP2C75, CYP2C76, and CYP2C93 mRNA. The expression level of each CYP2C mRNA was normalized to the 18S rRNA level and represents the average ± SD from three independent amplifications. (**A**) CYP2C93 mRNA expression was measured in cynomolgus monkey tissues, brain, lung, heart, liver, kidney, adrenal gland, jejunum, testis, ovary, and uterus. Among these tissues, CYP2C93 mRNA was predominantly expressed in liver. (**B**) Hepatic expression of CYP2C8, CYP2C43, CYP2C75, CYP2C76, and CYP2C93 mRNAs was measured in two rhesus monkeys expressing normal transcript CYP2C93 SV1. CYP2C93 mRNA was expressed at a lower level than other CYP2C mRNAs, but the difference in expression levels of CYP2C93 mRNA and other CYP2Cs varied in the two animals. CYP2C18 mRNA was excluded from the analysis due to its hepatic expression level substantially lower than other CYP2C mRNAs [16].

To analyze a tissue expression pattern of CYP2C93 mRNA, real-time RT-PCR was performed with gene-specific primers and probe using total RNAs prepared from cynomolgus monkey tissues, brain, lung, heart, liver, kidney, adrenal gland, jejunum, testis, ovary, and uterus. The experiment was not carried out in rhesus monkey due to the unavailability of the tissues. CYP2C93 mRNA was predominantly expressed in the liver, similar to cynomolgus monkey CYP2C8, CYP2C43, CYP2C75, and CYP2C76 mRNAs [6], with some extra-hepatic expression in brain and testis (Fig. 4A). To determine the expression level of CYP2C93 mRNA relative to other CYP2C mRNAs, CYP2C93 mRNA, along with CYP2C8, CYP2C43, CYP2C75, and CYP2C76 mRNAs, was measured in the livers of two rhesus monkeys expressing CYP2C93 SV1. CYP2C18 mRNA was excluded from the analysis due to its hepatic expression level substantially lower than other CYP2C mRNAs [16]. The analysis indicated that the expression level of CYP2C93 mRNA was lower than that of any other CYP2C mRNA (Fig. 4B). The expression level of each CYP2C mRNA varied between the rhesus monkeys; the expression level of CYP2C93 mRNA, in one animal, was only 2.1, 4.6, and 2.6-fold lower than that of CYP2C8, CYP2C43, and CYP2C75 mRNAs, respectively. In the rhesus monkeys (animal nos. 4, 5, 6, 8, and 10) that did not show a visible CYP2C93 band in the gel (Fig. 1B), CYP2C93 mRNA was actually expressed, but at substantially lower level, as determined by real-time RT-PCR (data not shown). CYP2C93 mRNA expression of rhesus monkeys was only 1.4-fold higher (approximately) in males than in females. These results suggest that CYP2C93 mRNA expression level is in general lower than other CYP2C mRNAs in rhesus monkey liver and is highly variable between the animals.

### Drug-metabolizing capability of CYP2C93 proteins

Enzymatic properties of cynomolgus and rhesus monkey CYP2C93, along with cynomolgus monkey CYP2C8, CYP2C43, CYP2C75, and CYP2C76, were measured using human CYP2C substrates, diclofenac, flurbiprofen, paclitaxel, *S*-mephenytoin, and tolbutamide. Cynomolgus monkey CYP2C93 proteins, mfCYP2C93v1 and mfCYP2C93v2, were prepared using the cynomolgus monkey CYP2C93 SV1 and SV2 expression plasmids, respectively. Since these cDNAs contained c.102T>del, c.102 was filled in with thymine by site-directed mutagenesis. Rhesus monkey CYP2C93 protein, mmCYP2C93v1, was prepared using the expression plasmid of the rhesus monkey CYP2C93 SV1 cDNA. The analysis by CO difference spectra indicated that a peak characteristic of P450 protein was seen around 450 nm for mfCYP2C93v1 and mmCYP2C93v1, but not for mfCYP2C93v2 (data not shown). mfCYP2C93v1 and mmCYP2C93v1 substantially catalyzed diclofenac 4-hydroxylation, flurbiprofen 4-hydroxylation, *S*-mephenytoin 4-hydroxylation, and tolbutamide methylhydroxylation, but not paclitaxel 6α-hydroxylation (Table 3), indicating a broad substrate selectivity of CYP2C93, unlike human and other macaque CYP2Cs. Although these reactions were also catalyzed by other CYP2Cs (diclofenac 4-hydroxylation and flurbiprofen 4-hydroxylation by CYP2C75, *S*-mephenytoin 4-hydroxylation by CYP2C43, and tolbutamide methylhydroxylation by CYP2C75 and CYP2C76), CYP2C93 showed a level of catalytic activity comparable to other CYP2Cs, indicating the involvement of CYP2C93 in CYP2C-mediated drug metabolism. mfCYP2C93v2 did not show an appreciable level of activity (Table 3), suggesting that CYP2C93v2 is nonfunctional. For the reactions catalyzed by CYP2C93, kinetic analysis was carried out using recombinant CYP2C93 protein (mmCYP2C93v1). For tolbutamide methylhydroxylation, diclofenac 4-hydroxylation, and flurbiprofen 4-hydroxylation, *K*_m_ was 1600±740, 15±5, and 260±34 µM, respectively, while *V*_max_ was 10.3±1.8, 0.27±0.02, and 0.13±0.01 nmol/min/nmol P450, respectively (Table 4). Kinetic analysis was also carried out for *S*-mephenytoin 4-hydroxylation, but parameters could not be determined due to low activity of CYP2C93 for this reaction.

**Table 3:** Drug-metabolizing activity of CYP2C93 protein determined using human CYP2C substrates.

P450	P450 content	CPR content	Paclitaxel 6α-hydroxylation	Tolbutamide methyl hydroxylation	Diclofenac 4′-hydroxylation	Flurbiprofen 4-hydroxylation	*S*-mephenytoin 4′-hydroxylation
	µM	µM	nmol/min/nmol P450	nmol/min/nmol P450	nmol/min/nmol P450	nmol/min/nmol P450	nmol/min/nmol P450
**Cynomolgus:**							
CYP2C8	30	5.5	0.26	0.41	<0.01	0.01	0.03
CYP2C43	8.5	3.1	<0.001	0.73	0.01	0.03	0.35
CYP2C75	19	7.8	<0.001	4.16	0.10	1.32	0.01
CYP2C76	13	3.4	<0.001	2.01	<0.01	0.01	<0.01
CYP2C93v1	2.3	1.6	<0.001	1.99	0.11	0.10	0.69
CYP2C93v2	<0.5	2.6	N.D.	<0.01	<0.01	<0.01	<0.01
**Rhesus:**							
CYP2C93v1	2.1	3.2	<0.001	3.25	0.38	0.76	0.16

CPR, cytochrome P450 reductase. N.D., not determined.

In each reaction, 5 pmol of the recombinant protein was used with substrate (50 µM diclofenac, 100 µM flurbiprofen, 100 µM paclitaxel, 200 µM *S*-mephenytoin, or 1 mM tolbutamide) as described in *Materials and Methods*.

The recombinant cynomolgus and rhesus monkey CYP2C93 proteins were analyzed along with cynomolgus monkey CYP2C8, CYP2C43, CYP2C75, and CYP2C76. CYP2C93v1 and CYP2C93v2 correspond to SV1 and SV2 transcripts of CYP2C93, respectively.

**Table 4:** Kinetic analysis for oxidations of typical human CYP2C9 substrates catalyzed by monkey CYP2C93.

Reaction	*K*_m_(µM)	*V*_max_(nmol/min/nmol CYP2C93)
Tolbutamide methyl hydroxylation	1600±740	10.3±1.8
Diclofenac 4′-hydroxylation	15±5	0.27±0.02
Flurbiprofen 4-hydroxylation	260±34	0.13±0.01

Each substrate (0–5000 µM tolbutamide, 0–200 µM diclofenac, and 0–200 µM flurbiprofen) was incubated with recombinant CYP2C93v1 (of rhesus monkey) at 37°C for 15 min in the presence of an NADPH-generating system as described in *Materials and Methods*. Kinetic parameters were calculated from a fitted curve by non-linear regression (mean ± SE).

### Analysis of *CYP2C93* IVS2-1G>T

**Figure 5:**
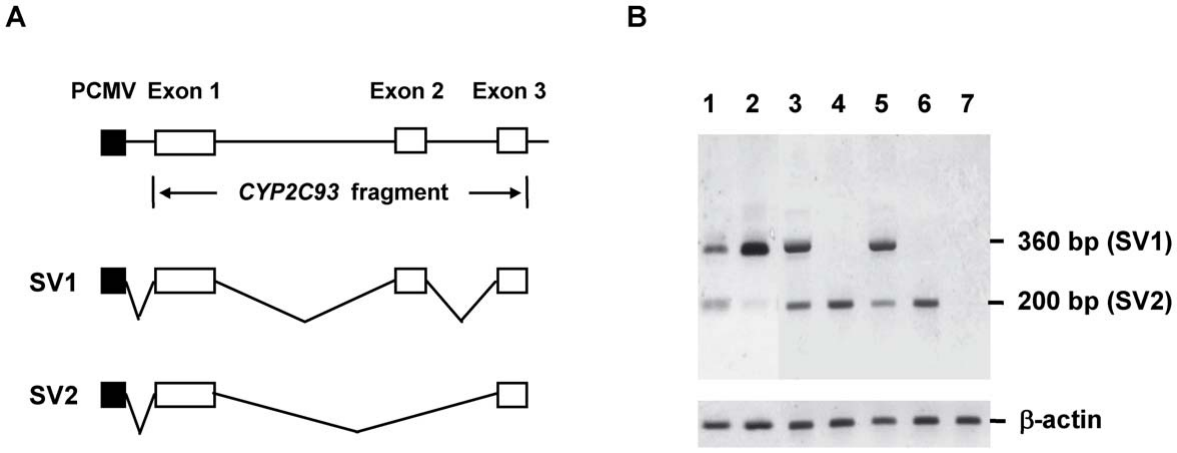
Mini gene assay for exon-2 skipping of CYP2C93. Preparation of expression plasmids and *in vitro* splicing analysis were performed as described in *Materials and Methods*. (**A**) Schematic illustration of the *CYP2C93* gene fragments (exon 1 to exon 3) used to generate mini gene plasmids. (**B**) *In vitro* splicing analysis. Each expression plasmid was transfected into COS1 cells, from which total RNA was extracted and used for amplification of CYP2C93 cDNA (from exon 1 to exon 3) by RT-PCR. The amplified products were visualized on an agarose gel. Lanes 1 and 2, liver total RNAs of the cynomolgus monkey (mfF1) expressing both SV1 and SV2 and the rhesus monkey (mm35) expressing SV1, respectively; lanes 3 and 4, IVS2-1G and IVS2-1T of cynomolgus monkey CYP2C93, respectively; lanes 5 and 6, IVS2-1G and IVS2-1T of rhesus monkey CYP2C93, respectively; and lane 7, mock. The upper band (360 bp) and lower band (200 bp) correspond to CYP2C93 SV1 and SV2 transcripts, respectively. For cynomolgus monkey and rhesus monkey CYP2C93, only exon-2 lacking SV2 was transcribed in the presence of IVS2-1T, whereas both SV1 and SV2 were transcribed in the presence of IVS2-1G. β-actin was also analyzed as a control.

To investigate exon-2 skipping of CYP2C93 SV2, the genomic region around exon 2 was sequenced for cynomolgus monkey (mfF1), expressing CYP2C93 SV1 and SV2, and for rhesus monkey (mm35), expressing only CYP2C93 SV1 (Fig. 5A). The comparison of the sequences found IVS2-1G>T only in cynomolgus monkey (mfF1, heterozygote), leading to the alteration of AG to AT at the splice site of intron 1. To investigate if IVS2-1G>T is responsible for exon-2 skipping; a mini gene assay was performed using the four expression plasmids containing the *CYP2C93* gene fragment from exon 1 to exon 3. Two plasmids (pcDNA3.1-mfCYP2C93_IVS2-1G and pcDNA3.1-mfCYP2C93_IVS2-1T) contained the *CYP2C93* gene fragment of cynomolgus monkey (mfF1), with IVS2-1G and IVS2-1T, respectively. The other two plasmids (pcDNA3.1-mmCYP2C93_IVS2-1G and pcDNA3.1-mmCYP2C93_IVS2-1T) contained the *CYP2C93* gene fragment of rhesus monkey (mm35), with IVS2-1G and IVS2-1T, respectively. Each plasmid was transfected into COS1 cells. RT-PCR using the total RNA extracted from the COS1 cells showed that CYP2C93 SV1 and SV2 (shown as a 360-bp upper band and a 200-bp lower band, respectively) were transcribed from the plasmids containing IVS2-1G, whereas only CYP2C93 SV2 was transcribed from the plasmids containing IVS2-1T (Fig. 5B). These results suggest that IVS2-1G>T was, at least partly, responsible for exon-2 skipping and generation of CYP2C93 SV2.

### Genotyping

The results described thus far showed that mfF1 (but not mm35) was heterozygous for IVS2-1G>T and c.102T>del. To see if other animals possess IVS2-1G>T and c.102T>del in *CYP2C93*, genotyping was performed for the animals (7 cynomolgus and 10 rhesus monkeys) analyzed by RT-PCR for expression of SV1 and SV2 (Fig. 1B). Among these animals, all the 7 cynomolgus monkeys were homozygous for IVS2-1G>T, whereas only one rhesus monkey possessed IVS2-1G>T (as a heterozygote) (Fig. 1B), indicating that IVS2-1G>T is prevalent in cynomolgus monkeys, but not in rhesus monkeys. Expression of SV2 in rhesus monkey (no. 3) and cynomolgus monkeys (nos. 1-7) appeared to be associated with IVS2-1T. None of the animals analyzed possessed c.102del, indicating that c.102T>del is a relatively rare allele possessed by only one cynomolgus monkey (mfF1) (as a heterozygote) in this study.

## Discussion

We previously identified CYP2C76, which is not orthologous to any human P450 and is at least partly responsible for the difference in drug metabolism between cynomolgus monkey and human [8]. Such a P450, if any, might also account for the species difference in macaques. In this study, we successfully identified a novel P450, CYP2C93 that had only 77–79% amino acid sequence identity to human CYP2Cs and an expression pattern, preferential to liver, which is similar to other CYP2Cs. Phylogenetic analysis indicated that CYP2C93, similar to CYP2C76, did not have a 1-to-1 relationship to any human CYP2C.

The lack of an orthologous relationship of CYP2C93 to human CYP2Cs was further supported by analysis of the genomic organization of *CYP2Cs*. *CYP2C93* was located, adjacent to *CYP2C76*, at the end of the *CYP2C* cluster; and, this position corresponds to the intergenic region in the human genome, indicating that a gene orthologous to *CYP2C93* does not exist in human. Genes in the P450 subfamilies including *CYP2Cs* have been generated by gene duplication during evolution (Nelson et al., 2004). A higher sequence identity of CYP2C93 to CYP2C8 as compared with other CYP2Cs, the position of CYP2C8 nearest to CYP2C93 in the phylogenetic tree, and the location of *CYP2C93* adjacent to *CYP2C8* in the *CYP2C* cluster, indicate that *CYP2C93* might have been generated as the result of gene duplication of a *CYP2C8*-like gene of a primate ancestor during evolution. Further refinement and assembly of genomic sequences in the macaque *CYP2C* cluster could lead to identification of additional gene(s) in this region.

CYP2C93 substantially catalyzed diclofenac 4′-hydroxylation, *S*-mephenytoin 4-hydroxylation, and tolbutamide methylhydroxylation. Diclofenac 4′-hydroxylation, *S*-mephenytoin 4-hydroxylation, and tolbutamide methylhydroxylation are catalyzed by rhesus monkey CYP2C75 [4], [17], cynomolgus monkey and rhesus monkey CYP2C43 [4], [5], [6], and cynomolgus monkey CYP2C75 and CYP2C76 [6], [9], respectively. Moreover, CYP2C93 metabolized the typical human CYP2C9 (diclofenac, flurbiprofen, and tolbutamide) and CYP2C19 (*S*-mephenytoin) substrates. Therefore, substrate selectivity of CYP2C93 appears to be broader than human and other monkey CYP2Cs. The specific substrates remained to be found for CYP2C93, which can partly ascertain the relevance of this enzyme to drug metabolism.

Diclofenac 4′-hydroxylation was largely catalyzed by CYP2C75 and CYP2C93 in this study. Previous study showed that *K*_m_ of rhesus monkey CYP2C75 was 15.8 µM (Tang et al.), similar to that of CYP2C93 (15 µM), indicating that the affinity to diclofenac is comparable between CYP2C75 and CYP2C93. Tolbutamide methylhydroxylation was catalyzed by CYP2C75, CYP2C76, and CYP2C93 in this study. *K*_m_ of cynomolgus monkey CYP2C75 and CYP2C76 was 775 and 886 µM, respectively (Uno et al., 2007), lower than that of CYP2C93 (1600 µM), indicating the lower affinity of tolbutamide to CYP2C93. Further investigation on kinetic analysis of various CYP2C substrates will lead to the better understanding of CYP2C93 enzyme property.

Paclitaxel 6α-hydroxylation is catalyzed by human CYP2C8. Among cynomolgus monkey CYP2Cs, only CYP2C8 substantially catalyzed this reaction, indicating that paclitaxel 6α-hydroxylation could be used as a probe reaction for CYP2C8 in cynomolgus monkey, similar to human. Although CYP2C93 had the highest sequence identity to CYP2C8 among the five cynomolgus monkey CYP2Cs, CYP2C93 did not catalyze paclitaxel 6α-hydroxylation. Similarly, marmoset CYP2C8, highly identical to human CYP2C8, does not metabolize paclitaxel, partly because the shape of the active site cavity for paclitaxel in marmoset CYP2C8 might be different from that of human CYP2C8 [18]. Similarly, CYP2C93 might possess an active site cavity different from human and cynomolgus monkey CYP2C8. It is of great interest to investigate the conformation of CYP2C93.

In two rhesus monkeys expressing normal CYP2C93 transcript (SV1), CYP2C93 mRNA was expressed at a lower level than other CYP2C mRNAs; however, in one animal the difference of CYP2C93 in mRNA expression level was only 2.1- and 2.6-fold as compared with CYP2C8 and CYP2C75 mRNAs, respectively. In contrast, 5 of the 11 rhesus monkeys analyzed expressed CYP2C93 mRNA at greatly lower level in liver, indicating that CYP2C93 mRNA expression is highly variable in rhesus monkeys. Moreover, all the 8 cynomolgus monkeys analyzed, expressed only aberrantly spliced SV2 (nonfunctional) or expressed nonfunctional SV1 due to another mutation c.102T>del. These results suggest that a functional CYP2C93 is expressed in rhesus monkeys, but not in cynomolgus monkeys. Therefore, the species and the animals should be carefully selected for drug metabolism studies using monkeys. The importance of aberrant splicing in drug metabolism has been recognized in human. For example, expression of CYP3A5 is highly polymorphic between whites and African Americans; CYP3A5 protein is present in 10 to 30% of whites and 60% of African Americans, due to aberrant splicing [19], [20], [21]. In this study, the exon-2 skipping of the nonfunctional transcript SV2 was, at least partly, accounted for by a mutation at the splice site of intron 1, IVS2-1G>T, as indicated by mini gene assay. This genotype can be utilized to identify animals expressing a nonfunctional transcript SV2.

Although the differences in drug metabolism between two macaque species (cynomolgus monkey and rhesus monkey) has not been fully investigated, a previous study showed that *S*-mephenytoin 4-hydroxylation was higher in cynomolgus monkeys than in rhesus monkeys [22]. This is contrary to our expectation, considering that CYP2C93 protein, which also catalyzes this reaction, might be present in rhesus monkey, but not in cynomolgus monkey, as shown in this study. Alternatively, the difference in *S*-mephenytoin 4-hydroxylation between the two macaque species can be accounted for by the variability in CYP2C43 metabolic properties as well, since CYP2C43 also catalyzes this reaction. Such variability in drug-metabolizing activities could be partly owing to genetic divergence in *CYP2C43*, as genetic polymorphisms are evident in macaque *P450s* [23], [24], similar to human *P450s*. The genetic polymorphisms also account for the inter-animal variability of drug-metabolizing enzyme activities. Inter-animal differences are important for studies using monkeys, since interpretation of the data could sometimes be complicated. Therefore, an investigation of genetic variants in drug-metabolizing enzyme genes is essential for successful drug metabolism studies using monkeys. To this end, again, IVS2-1G>T is useful to investigate, if any, species and inter-animal differences in CYP2C93-dependent drug metabolism.

CYP2C93 was successfully identified, based on rhesus monkey genome data. As genome data such as genome sequences and EST have become increasingly available to the public, such genomic information has been applied to the identification of species-specific genes in monkeys [25], [26]. We also successfully utilized EST data to identify species-specific CYP2C76, which does not correspond to any human P450s and is partly responsible for species differences in drug metabolism between monkey and human [8], [27]. This paper further supports the usefulness of genome information. As the throughput of sequence generation increases greatly with next-generation sequencers, the more genome information becomes available, and can be utilized to better understand various biological events of physiology and disease, including drug metabolism.

In conclusion, we identified macaque CYP2C93, which is not orthologous to any human P450s, based on sequence and genome analysis. This initial study showed that CYP2C93 mRNA was predominantly expressed in the liver and that CYP2C93 protein was a functional enzyme, metabolizing human CYP2C substrates (diclofenac, flurbiprofen, paclitaxel, *S*-mephenytoin, and tolbutamide). A nonfunctional transcript variant (SV2) lacking exon 2 was generated partly due to the mutation at the splice site in *CYP2C93* intron 1. The functional transcript variant (SV1) was found in 6 of the 11 rhesus monkeys, but not in all the 9 cynomolgus monkeys analyzed. Lower expression of CYP2C93 mRNA than other CYP2C mRNAs in rhesus monkey liver, and broad substrate specificity of CYP2C93, suggest that CYP2C93 might play minor roles in drug metabolism. However, this does not preclude the potential relevance of rhesus monkey CYP2C93, depending on the animal, to metabolism of the drugs, if any, that are mediated specifically by CYP2C93.
